# Cost-benefit analysis of a population-based education program on the wise use of antibiotics

**DOI:** 10.17269/s41997-019-00245-w

**Published:** 2019-08-16

**Authors:** Abdullah Mamun, Bin Zhao, Mark McCabe, Kim Dreher, Michael Otterstatter, Nick Smith, Edith Blondel-Hill, Fawziah Marra, David M. Patrick

**Affiliations:** 1 University of British Columbia, Vancouver, BC Canada; 2 BC Centre for Disease Control, Provincial Health Services Authority, Vancouver, BC Canada; 3 Interior Health Authority, Kelowna, BC Canada

**Keywords:** Antibiotics, Education, Population, Time series, Cost, Antibiotiques, Éducation, Population, Série chronologique, Coût

## Abstract

**Objective:**

In 2005, the Do Bugs Need Drugs (DBND) program was imported to British Columbia (BC) from Alberta with the goal of reducing unnecessary antibiotic use in the community. The objective of this study was to estimate the impact of the program on antibiotic-associated costs and cost-benefit.

**Methods:**

We used data on antibiotic prescription and costs from BC PharmaNet for the period of 1996 to 2014. We conducted interrupted time series regression to formally interpret the impact of the DBND program.

**Results:**

The average monthly prescription rate fell by 14.5%, from 54.3 to 46.4 per 1000 population between 2005 and 2014. The proportionate contribution of macrolide prescription decreased from 19.2% in 2005 to 13.2% in 2014 and for quinolones decreased from 13.1% in 2005 to 12% in 2014. The proportion of prescriptions for both penicillins and tetracyclines increased by > 35.5%. Before the program, the average monthly cost of antibiotics was increasing by CAD $8.12 per 1000 population (*p* < 0.001). After program introduction, average monthly cost decreased by CAD $18.19 per 1000 population (*p* < 0.001), creating an annual savings for BC in 2014 of CAD $83.6 million. In 2014, one Canadian dollar spent on the DBND program was associated with conservative savings of CAD $76.20.

**Conclusion:**

Significant cost savings have been observed in association with a community antimicrobial stewardship program focused on both public and prescribers. Such programs are an effective strategy in cost-benefit terms and should therefore be considered for universal adoption in Canadian healthcare systems.

## Résumé

**Objectif:**

En 2005, la Colombie-Britannique a importé de l’Alberta le programme Do Bugs Need Drugs (DBND) en vue de réduire l’utilisation non nécessaire des antibiotiques dans la population. L’objectif de notre étude était d’estimer les incidences du programme sur les coûts et le rapport coûts-avantages associés aux antibiotiques.

**Méthode:**

Nous avons utilisé les données de BC PharmaNet sur les ordonnances d’antibiotiques et les coûts des antibiotiques pour la période de 1996 à 2014. Nous avons mené une régression par série chronologique interrompue pour interpréter officiellement les incidences du programme DBND.

**Résultats:**

Le taux d’ordonnances mensuel moyen a baissé de 14,5 %, soit de 54,3 à 46,4 pour 1 000 habitants entre 2005 et 2014. Le poids proportionnel des ordonnances de macrolides a baissé, passant de 19,2 % en 2005 à 13,2 % en 2014; celui des quinolones a également baissé, de 13,1 % en 2005 à 12 % en 2014. Le poids des ordonnances de pénicillines et de tétracyclines a augmenté de plus de 35,5 %. Avant le programme, le coût moyen des antibiotiques augmentait de 8,12 $CAN pour 1 000 habitants par mois (*p* < 0,001). Après l’introduction du programme, le coût moyen a diminué de 18,19 $CAN pour 1 000 habitants par mois (*p* < 0,001), ce qui a permis à la Colombie-Britannique d’économiser 83,6 M $CAN pour l'année 2014. En 2014, un dollar canadien dépensé pour le programme DBND était associé à une économie d'au moins 76,2 $CAN.

**Conclusion:**

D’importantes économies de coûts ont été observées en lien avec un programme collectif d’intendance des antimicrobiens axé à la fois sur le grand public et sur les médecins prescripteurs. De tels programmes représentent une stratégie efficace du point de vue du rapport coûts-avantages; il faudrait en envisager l’adoption universelle dans les systèmes canadiens de soins de santé.

## Introduction

Antimicrobial resistance (AMR) is a significant threat to health and to healthcare system sustainability (O’Neill 2014). An increase in AMR could threaten the safety of surgery, cancer treatment and immunosuppressive strategies and could cost trillions by 2050 (O’Neill 2016). Development of new antibiotics and alternatives is part of the solution, but very few new compounds are currently coming to market. The World Health Organization (WHO) has recommended other areas for action that have been adopted by many nation states (World Health Organization 2015). These include improving surveillance of AMR and antimicrobial use, enhancing efforts in infection prevention and control and investing in antimicrobial stewardship.

Most efforts in antibiotic stewardship have focused on hospital and institutional care, but there are few examples of programs aimed at addressing the 80–90% of human antibiotic use that results from community prescriptions. A systematic review has concluded that public-facing programs that address both general public and clinicians can reduce antibiotic use (Cross et al. 2017).

Given the strong links between antimicrobial use and resistance at the population level (Goossens et al. 2005), the Do Bugs Need Drugs (DBND) program was imported into British Columbia (BC), Canada, from the neighbouring province of Alberta in 2005. DBND is an innovative program of community and provider education aimed at reducing unnecessary antibiotic use. Program components include freely accessible guidelines and continuing health education for prescribers, direct outreach through schools, daycares and community care facilities using a workforce of volunteer health sciences students and pharmacists, and public campaigns ranging from transit ads to social media (Table 1). The province of BC has 4.6 million people in an area nearly three times that of Germany and more than twice the size of California. Initiation of various program components varied but was more or less complete by 2009.

**Table 1:** Do Bugs Need Drugs program activities with duration of implementation

Activity	Duration^a^
Program media launch	2005–2006
TV advertising	2006–2014
Typically January/February	
Transit advertising	2007–2014
Typically September–November	
Radio advertising	2007–2010
Children’s outreach and advertising	2008–2010
Including children’s magazine educational inserts, Vancouver International Children’s Festival sponsorship, children’s website advertising	
Grade 2 DBND curriculum	2006–2014
Daycare DBND curriculum	2006–2014
General teaching DBND curriculum	2006–2014
Older adult DBND curriculum	2008–2014
Kindergarten–Grade 3 DBND teaching resources	2010–2014
Educational lectures and workshops for healthcare practitioners and students	2006–2014
Information print material distribution	2005–2014
Do Bugs Need Drugs website	2005–2014
Antibiotic Wise website	2014
Bugs & Drugs reference guide distribution	2006–2014
Began as hardcopy distribution, then mobile app, now website	
Educational packages for pharmacists and physicians	2006
Parent-targeted advertising	2005, 2009
Movie theatre advertising	2005–2006
Peer-reviewed journal publication	2010–2014
Surveillance and reporting of AMR and AMU trends	2010–2014
Online and social media advertising	2014

*AMR* antimicrobial resistance, *AMU* antimicrobial use

^a^Some of the activities were implemented during different times of a year, not necessarily the whole year indicated within the table

After observing a decline in antibiotic use and crude costs since the program onset, we set out to better estimate the impact of the program itself taking into account key covariates such as population size and unit cost of drugs. Previous studies have focused on evaluation of institution-based antibiotic stewardship or clinical and microbiological outcomes after stewardship program implementation (Dik et al. 2015; Dik et al. 2016). But this study is different as it evaluates the effect of implementing a community-focused antibiotic stewardship program. The objective of this study was to examine the strength and magnitude of changes in antibiotic use and cost associated with DBND program implementation using interrupted time series analysis. Increasingly, time series analyses are advocated as a method to evaluate community interventions (Biglan et al. 2000).

## Methods

We obtained data on antibiotic use and costs from BC PharmaNet, a database that captures information on all outpatient prescriptions dispensed by community pharmacies in the Canadian province of British Columbia, with the exception of some drugs used for HIV and STI. Antibiotic prescription data for the period 1996 through 2014 were extracted by a third party within the BC Ministry of Health; patient identifiers were removed and we received an anonymized dataset. Records were line-listed by prescription and included fields on patient demographics, prescriber profession, date of prescription, and the name, dose, frequency and duration of therapy. We classified antibiotics according to the Anatomical Therapeutic Chemical (ATC) classification system developed by WHO (World Health Organization 1996). Annual provincial population estimates were obtained from the Government of BC Vital Statistics (BC Statistics 2018).

For the purposes of the time series analysis, we report antibiotic use in prescriptions per 1000 population per month. We analyzed the data first with respect to overall rate of antibiotic use, then according to use of the seven major ATC classes and the cost in Canadian dollars (CAD) monthly per 1000 population. For the monthly cost of the antibiotic, we calculated the total cost (i.e., cost of the medication and pharmacist dispensing fee); this cost was paid by either the government of BC (through PharmaCare insurance) and/or the patient (directly or through an additional extended benefits insurance plan).

Because community guidelines for many indications emphasize beta-lactam (J01C) and tetracycline (J01A) antibiotics as first-line agents, we looked for a change in the proportion of use attributed to these classes as evidence of class switching in alignment with the DBND program goals.

Over the course of the study period, a number of factors external to the DBND program could have influenced cost. We adjusted for these as follows: population adjustment was achieved by our focus on rates per population in our models; we adjusted for unit drug price change by setting the average price per unit for each drug (both generic and brand) in each year to their corresponding average price in 2014. For those drugs not present in 2014, we calculated the average price per unit of drug *x* in year *y* using the formula: (average drug price per unit of all drugs in 2014)/(average drug price per unit of all drugs in year *y*) × (average drug price per unit of drug *x* of that drug in year *y*). Since one of the objectives of this study was to examine the changes in antibiotic costs associated with DBND program implementation, adjusting for unit drug price took care of both inflation and changes in real prices over time.

To assess the impact of the DBND program, we conducted an interrupted time series analysis based on changes in monthly total cost for antibiotics per 1000 population. We first removed seasonal trends from the raw cost data, and then determined the time series components to include in our regression model, and finally fit a regression model including terms for the DBND program. We began by examining the data prior to the program (January 1, 1996 to August 31, 2005) and after its initiation (September 1, 2005 to December 31, 2014), looking for overall and seasonal trends in the yearly and monthly rates. We removed any seasonal trends using time series decomposition techniques (Electronic Statistics Textbook, StatSoft, Tulsa, OK, 2013) and focused on the seasonally adjusted data for all further analysis. An autoregressive moving average (ARMA) model was fit to determine appropriate autoregressive and moving average terms to include in our final analysis. We assessed stationarity and serial autocorrelation in the ARMA model using standard methods (i.e., augmented Dickey-Fuller test, autocorrelation and partial autocorrelation function plots of the seasonality adjusted data) (Chatfield 2016). The best time series components were determined using the model that minimized AIC. We also plotted the fitted model’s residuals to see the distribution of the observed error in order to check whether the fitted model is systematically correct or needs to be improved.

Our final analysis was a generalized least squares regression model of the seasonally adjusted cost data, including covariates for the time series (ARMA) components, the initiation of the DBND program and the duration of time since program initiation (Cowpertwait and Metcalfe 2009; Chatfield 2016). In addition to our main analysis, we also considered a more conservative measure of the program’s effect. We compared the observed mean monthly cost for antibiotics in 2014 to the mean monthly cost for antibiotics in the year just before program implementation (2005). This mean monthly conservative estimation of change in cost was then applied to the population of BC in 2014 to estimate the conservative cost reduction between the program start time and 2014. We also applied the final regression model separately for each of the five health authorities of BC to estimate regional variation in this conservative cost reduction. The total expenditures of the DBND program were calculated until December 2014 and were compared to the extrapolated conservative total cost reduction for antibiotics, to estimate the reduction/saved against a dollar spent for the DBND program (cost-benefit analysis).

All analyses were performed using SAS 9.4 (SAS Institute, Cary, North Carolina) and R version 3.4.1, with a *p*-value less than 0.05 defined as statistically significant.

## Results

**Fig. 1:**
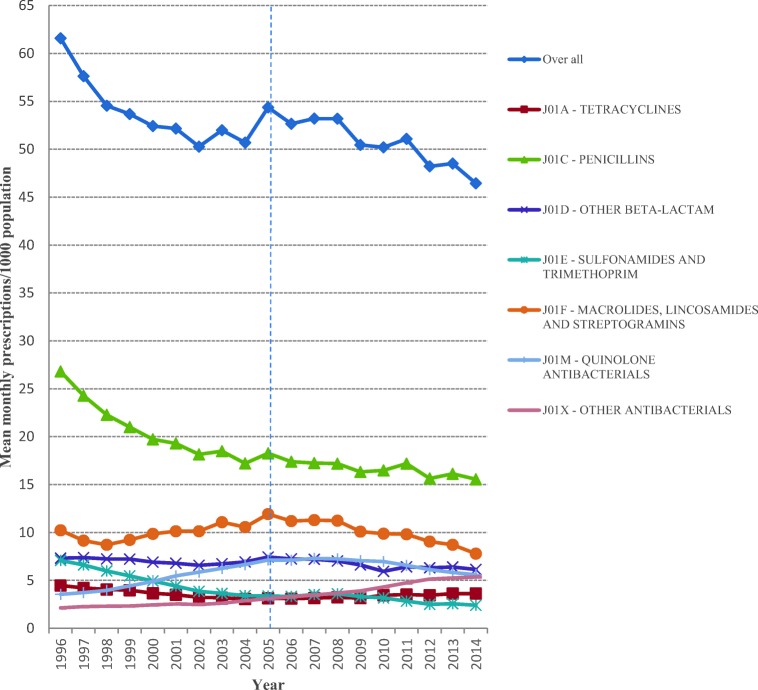
Mean monthly prescriptions per thousand population in BC, 1996–2014

**Fig. 2:**
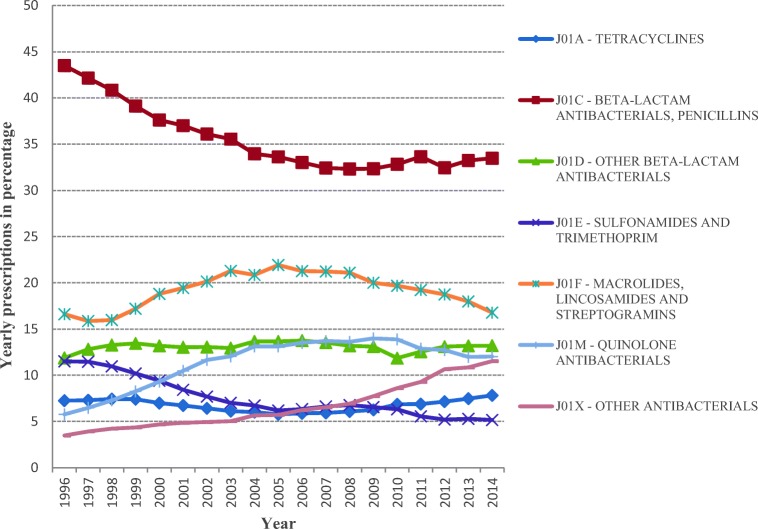
Yearly proportion of prescriptions in BC, 1996–2014

The overall average monthly prescription rates ranged from 54.3 per 1000 population in 2005 to 46.4 per 1000 population in 2014, a reduction of 14.5%. In line with the overall decrease in prescription rates, mean monthly prescription rates decreased for quinolones, macrolides and sulfonamides and trimethoprim antibiotics (Fig. 1). Decreases were also observed in the yearly proportion that each drug comprised of the total prescriptions with the exception of tetracyclines and penicillins, which increased in their prescription rates by about 15% from 2005 to 2014 and in their yearly proportions by 35.6% and 0.4%, respectively (Fig. 2).

**Fig. 3:**
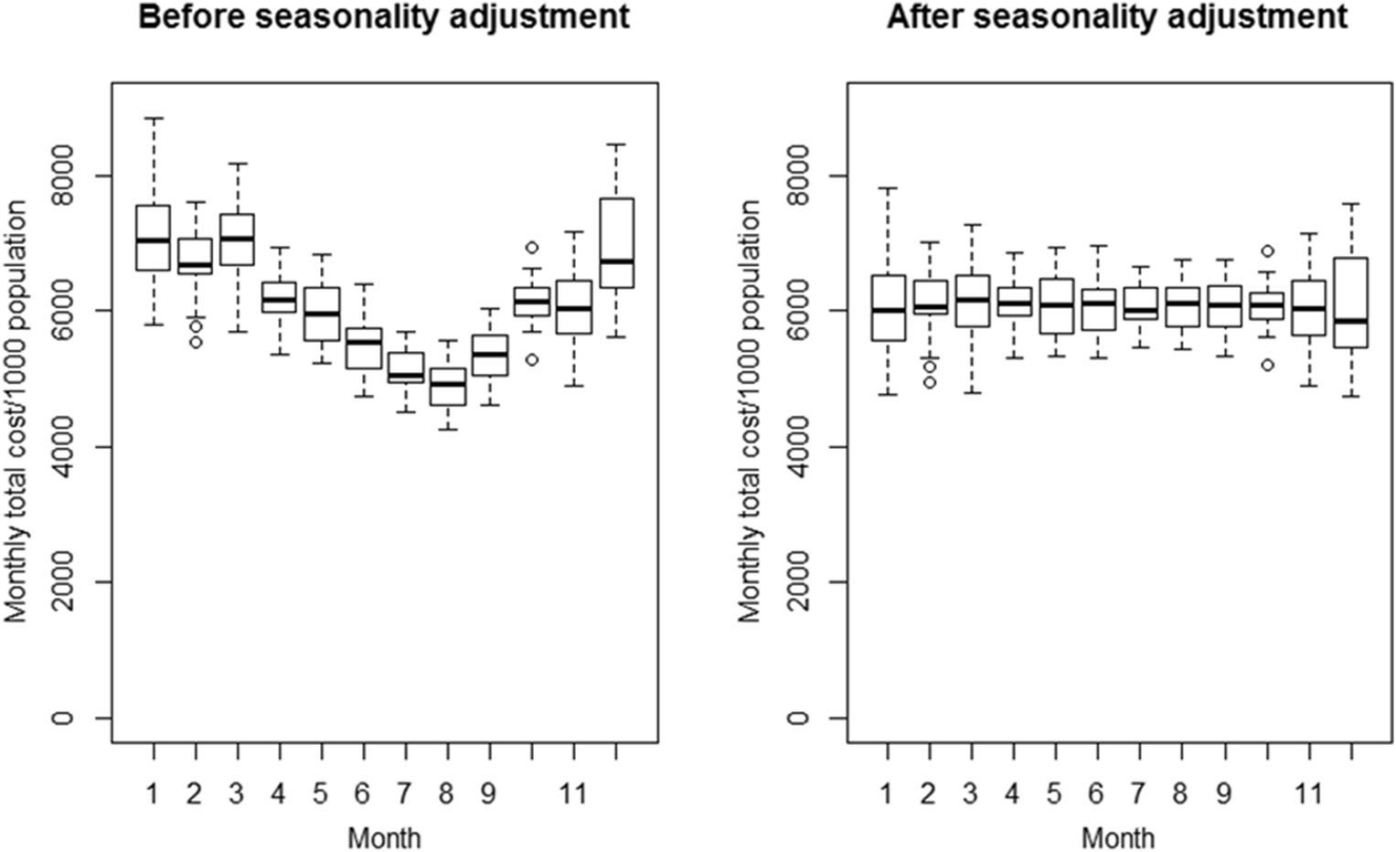
Boxplot showing median cost of antibiotics in BC by calendar month before and after seasonality adjustment, 1996–2014

The mean monthly costs of antibiotics (adjusted for drug unit cost) per 1000 population both before and after seasonal adjustment are displayed in Fig. 3. As expected, unadjusted mean monthly costs were highest between December and March.

Before modeling, the crude reduction in average monthly cost of antibiotics (adjusted for drug unit cost) fell by $1,465 CAD between 2005 and 2014, representing a reduction in total annual expenditures on antibiotics of $80.8 million CAD. Sixty-six percent of this cost savings was realized by patients or their third-party benefits insurer and the remainder was realized by PharmaCare (BC’s government-funded pharmaceutical plan).

### Interrupted time series analysis

**Fig. 4:**
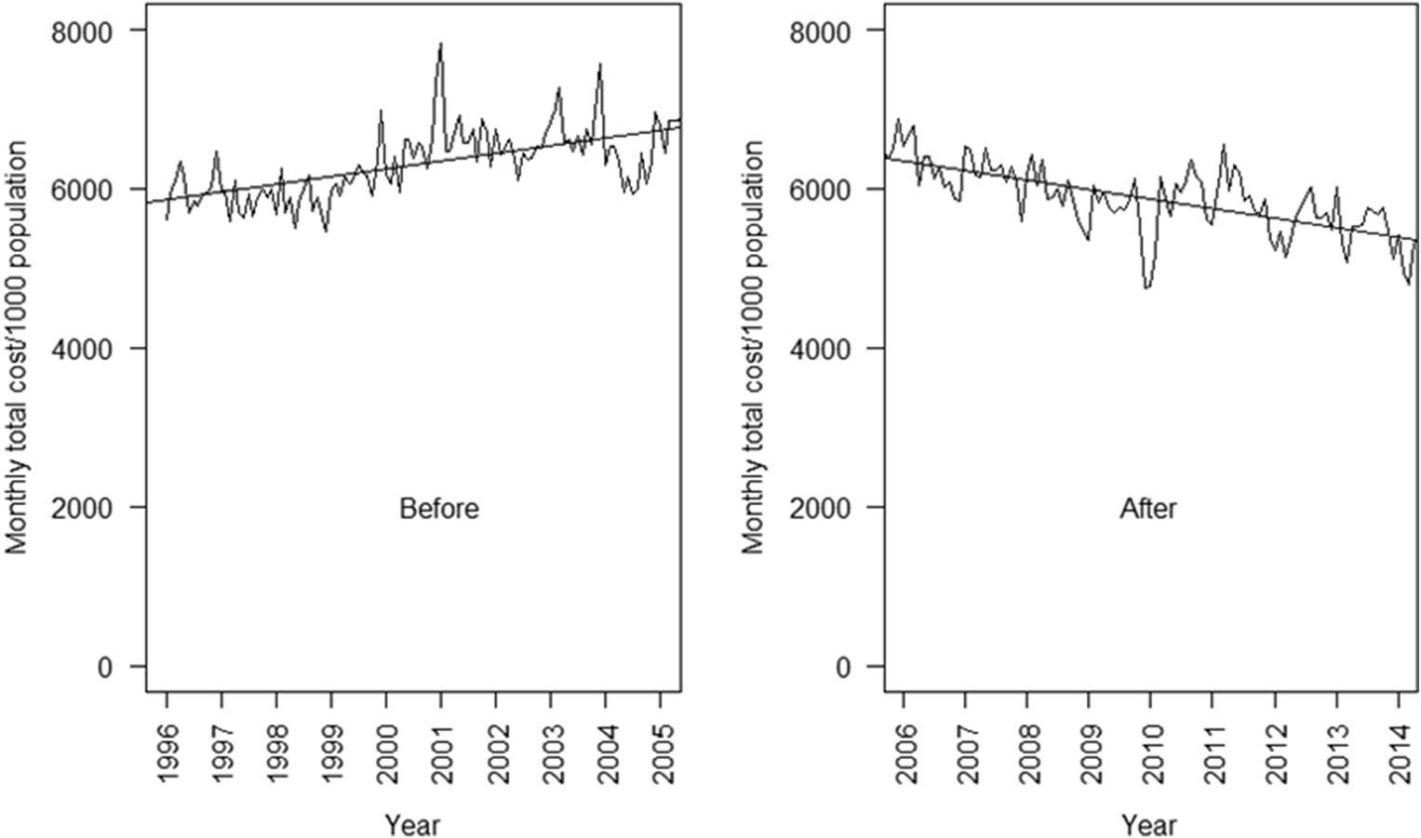
Trends in monthly total cost of antibiotics before and after the DBND program in BC, 1996–2014

The total monthly cost for antibiotics was increasing before the start of the DBND program, but then declined steadily following program implementation (Fig. 4). Table 2 shows the parameter estimates from the segmented regression model of the effects of the DBND program on mean monthly total cost of antibiotics per 1000 population. The results of the regression indicate that before implementation of the program, the mean monthly total cost for antibiotics was $5845.39 CAD/1000 population and there was a significant month to month increase ($8.12 CAD/1000 population) in the total cost (*p* < 0.0001). There was a significant drop in the estimated mean monthly total cost (coefficient = − 384.98, *p* = 0.001) after the DBND program began (Table 2). In addition to this level change, there was a significant change in the trend, which began to decline after the program by a monthly average of $18.20 CAD in total cost of antibiotics per 1000 population (*p* < 0.0001). The regression models also show a statistically significant month to month change in total cost of antibiotics both for the level and trend for each of the five health authorities in BC (Table 3).

**Table 2:** Segmented regression model estimating the parameters and predicting mean monthly total cost per 1000 population over time in BC, 1996–2014

Components/variables	Estimate ($)	Standard error ($)	t-statistic	*P* value
Intercept	5845.39	82.79	70.60	< 0.0001
Baseline trend	8.12	1.22	6.64	< 0.0001
Level change after intervention	− 384.98	115.60	− 3.33	0.001
Trend change after intervention	− 18.19	1.36	− 10.15	< 0.0001

**Table 3:** Segmented regression model estimating the parameters for health authorities in BC, 1996–2014

	Fraser Health Authority	Vancouver Coastal Health Authority	Vancouver Island Health Authority	Interior Health Authority	Northern Health Authority
Components	Estimate ($)	Estimate ($)	Estimate ($)	Estimate ($)	Estimate ($)
Intercept^a^	5845.3	5981.6	5651.6	5492.03	5495.4
Baseline trend^b^	8.1	4.29	13.01	3.42	− 3.93
Level change after intervention^c^	− 384.9	− 269.7	− 802.2	− 372.10	− 313.26
Trend change after intervention^d^	− 24.6	− 16.8	− 21.3	− 9.4	− 4.4

^a^Estimates are significant (*p* < 0.001), ^b^ estimates are significant (*p* < 0.001)

^c^Estimates are significant (*p* < 0.01), ^d^ estimates are significant (*p* < 0.01)

### Program effects

Using Table 2, we estimated that in December 2014, the mean monthly total cost of antibiotics was $5284.90 CAD per 1000 persons. Had the program not been introduced, the mean monthly total cost of antibiotics was on a course to reaching $7689.80 CAD/1000 persons by December 2014. This means that the mean monthly total cost of antibiotics decreased by a maximum of $2404.90 CAD, a 31% reduction after the DBND program implementation.

We also estimated the reduction in mean monthly cost of antibiotics more conservatively: as the difference between the observed mean monthly cost of antibiotics in 2014 and the model estimated mean monthly cost of antibiotics in the last month before program implementation (August 2005). This conservative estimation of the mean monthly total cost of antibiotics reduction per 1000 population in December 2014 was $1503 CAD (21% of mean monthly cost of antibiotics in August 2005) (Table 4). Of this reduction, the patient portion represented $933.80 CAD/month/1000 population, whereas the BC PharmaCare portion represented the remainder (Table 4). Based on the parameters in Table 3, we also estimated the conservative impact of the DBND program on 2014 antibiotic cost for each of the health authorities—ranging from $4.27 million CAD to $33.46 million CAD (Table 3).

**Table 4:** Changes in mean monthly costs per 1000 population after implementing the DBND program in BC, 2014

Costs	Changes in slope			Maximum^a^ estimated reduction	Conservative^b^ estimated reduction	Conservative impact on 2014 drug costs in millions in BC
	Mean	95% CI	*P* value	Mean (%)	Mean (%)	
Total ($)	− 18.2	− 21.7, − 14.6	< 0.001	− 2404.87 (− 31.3)	− 1503 (− 21.1)	83.6
PharmaCare cost ($)	− 3.3	− 8.0, 1.3	0.16	− 398.26 (− 29.4)	− 568.9 (− 37.3)	31.7
Patient cost ($)	− 15.4	− 19, − 11.8	< 0.001	− 1898.13 (− 30.8)	− 933.83 (− 18)	51.9

^a^Difference between the observed mean monthly cost of antibiotics and the expected mean monthly cost of antibiotics had the intervention not taken place

^b^Difference between the observed mean monthly cost of antibiotics in December 2014 and the mean monthly cost of antibiotics just before program implementation

### Cost analysis

Based on the conservative estimate of total monthly cost of antibiotics per 1000 population, we estimated a reduction of $83.6 million CAD for antibiotics in 2014. In the same way, we also estimated the reduction in PharmaCare cost and patient cost ($31.7 million CAD and $51.9 million CAD, respectively) for the year 2014. A total of $5.9 million CAD has been invested to implement the DBND program components in BC between the program start date and end of 2014. However, we estimated a total of $449.7 million CAD reduction in total cost of antibiotics during this time period. Using the total DBND program cost and the conservative estimation of total cost reduction for antibiotics, we estimated that every $1 CAD spent for the program saved a total of $76.20 CAD.

## Discussion

Our findings shed light on the potential value of population-based antimicrobial stewardship programs. This 19-year time series analysis indicates that the DBND program was successful in reducing overall antibiotic utilization and bringing about targeted class switching and that it was associated with significant antibiotic cost savings for BC, which does not necessarily determine causality. During 2006–2014, a total of $449.7 million CAD was saved, with patients and their third–party insurers realizing almost two thirds of the savings.

The estimated cost reduction for antibiotics reported in this study was conservative because we assumed that the total costs would have remained at the level observed just before program implementation. In fact, our analyses showed that costs were trending steadily upward prior to the program, in part due to undesirable class switching to new macrolides and fluoroquinolones. Our analysis did not include other potential societal cost savings, such as those attributable to adverse events due to antibiotic use and subsequent hospital visits and stays. Where possible, taking into account a more complete societal perspective would have given a more robust and greater cost savings in this study (Oppong et al. 2013; Coulter et al. 2015; Dik et al. 2015; Drummond et al. 2015). The effect of a stewardship program on costs aligns with studies reporting on institutional stewardship efforts (Coulter et al. 2015). However, this study represents one of the first evaluations of cost savings for community antibiotic stewardship at the population level.

In this study, we observed a gradual and continuous impact of the program on average total monthly cost reduction for antibiotics as evidenced by the slope/trend change after intervention. This is expected with a multifaceted community program rolled out gradually, as not all program components began simultaneously across all the health authorities in BC. We observed the total monthly cost reduction of antibiotics in individual health authorities within BC ranging from $4.2 to $33.4 million CAD in 2014. We estimated that one Canadian dollar spent for the DBND program implementation saved $76.20 CAD. Given this return on investment, most health jurisdictions or organizations should consider the value of community-based antibiotic stewardship efforts, as they show the promise of achieving better practice while also saving costs.

Observed cost reductions were driven both by a significant drop in monthly prescription rate and by antibiotic class switching toward drugs of first choice. A significant portion of total cost reduction of antibiotics can be attributable to the change in the usage of antibiotic classes; that is, increasing the usage of less costly penicillins and tetracyclines and reducing the usage of more expensive fluoroquinolones and newer macrolides.

There are some important limitations for our study. First, it is ideal to have a clear start and end point of an intervention in a time series analysis (Bernal et al. 2017). In this study, the program components rolled out at different time points and not simultaneously in all the health authorities of BC. In our case, the program had a clear start point but continued to run until the end of the study period. Had we included a “wash-in” period to represent program start-up, we would have elevated our estimate of cost savings, so have preferred to present the more conservative case.

While we reported the impact of the program, we assumed that the observed temporal changes in mean monthly cost of antibiotics and associated cost savings are due to the program. This is an assumption of the modeling approach and we acknowledge that the regression model cannot determine causality. We could not measure and adjust for other co-interventions that were in place during the study period which might have had an impact on the cost reductions in this study. For instance, introduction of pneumococcal conjugate vaccine (PCV) 7 in 2003 and PCV 13 in 2010 for infant and childhood vaccination could have reduced the burden of pneumococcal infections (Low 2017). However, one Canadian study reported that while incidence of invasive pneumococcal disease declined for children < 5 years, incidence was relatively unchanged in ages ≥ 5 years (Demczuk et al. 2013).

A common problem with population-level public health interventions is the inability to assign a control. However, because the time series data in this study had a pre-intervention segment that served as a control for the post-intervention segment, this should address the threat to internal validity (Wagner et al. 2002).

Programs with multifaceted educational components like DBND often diffuse gradually through the target population and the time to see an impact of such programs is related to the diffusion process and/or rate. This is why it is important to have the knowledge of diffusion process and diffusion rate after the onset of the program/intervention (Wagner et al. 2002). For this study, we hypothesized based on the history and maturation that the diffusion process will be gradual but we could not measure the diffusion rate of the program through the related population.

We did not measure the individual program component effect and so we are not certain which component of the program contributed most to the observed changes in this study. Often, it is difficult to calculate this type of measure where a series of efforts are involved in implementing a multifaceted community intervention (Biglan et al. 2000). However, based on the assumptions from the history and experiences during the program implementation, the combined DBND component(s) are presumed to be effective.

## Conclusion

Our findings showed that significant cost savings have been observed in association with a community antimicrobial stewardship program focused on both public and prescribers, even after conservative estimation. Such programs are an effective strategy in cost-benefit terms and should therefore be considered for universal adoption in Canadian healthcare systems. However, additional work needs to be done in regard to the appropriateness of prescribing antibiotics and any unintentional consequences of prescriptions reduction.

## References

[CR1] BC Statistics, Province of British Columbia (2018). Retrieved April 6, 2018, from https://www2.gov.bc.ca/gov/content/data/statistics/people-population-community/population/population-estimates.

[CR2] Bernal JL, Cummins S, Gasparrini A (2017). Interrupted time series regression for the evaluation of public health interventions: a tutorial. International Journal of Epidemiology.

[CR3] Biglan A, Ary D, Wagenaar AC (2000). The value of interrupted time-series experiments for community intervention research. Prevention Science.

[CR4] Chatfield, C. (2016). *The analysis of time series: an introduction*. CRC Press.

[CR5] Coulter S, Merollini K, Roberts JA, Graves N, Halton K (2015). The need for cost-effectiveness analyses of antimicrobial stewardship programmes: a structured review. International journal of antimicrobial agents.

[CR6] Cowpertwait P, Metcalfe A (2009). Introductory time series with R, 2009.

[CR7] Cross ELA, Tolfree R, Kipping R (2017). Systematic review of public-targeted communication interventions to improve antibiotic use. Journal of Antimicrobial Chemotherapy.

[CR8] Demczuk WHB, Martin I, Griffith A, Lefebvre B, McGeer A, Lovgren M, Tyrrell GJ, Desai S, Sherrard L, Adam H (2013). Serotype distribution of invasive Streptococcus pneumoniae in Canada after the introduction of the 13-valent pneumococcal conjugate vaccine, 2010–2012. Canadian Journal of Microbiology.

[CR9] Dik, J.-W. H., Vemer, P., Friedrich, A. W., Hendrix, R., Lo-Ten-Foe, J. R., Sinha, B., & Postma, M. J. (2015). Financial evaluations of antibiotic stewardship programs—a systematic review. *Frontiers in Microbiology, 6*. 10.3389/fmicb.2015.00317 PMC4399335 25932024

[CR10] Dik J-WH, Hendrix R, Poelman R, Niesters HG, Postma MJ, Sinha B, Friedrich AW (2016). Measuring the impact of antimicrobial stewardship programs. Expert Review of Anti-infective Therapy.

[CR11] Drummond MF, Sculpher MJ, Claxton K, Stoddart GL, Torrance GW (2015). Methods for the economic evaluation of health care programmes.

[CR12] Goossens H, Ferech M, Stichele RV, Elseviers M, E. P. Group (2005). Outpatient antibiotic use in Europe and association with resistance: a cross-national database study. Lancet.

[CR13] Low, A. (2017). "Pneumococcal immunization in the elderly and immunocompromised." Retrieved 14 May, 2018, from https://www.bcpharmacy.ca/news/pneumococcal-immunization-elderly-and-immunocompromised.

[CR14] O’Neill, J. (2014). Antimicrobial resistance: tackling a crisis for the health and wealth of nations. *Review on Antimicrobial Resistance*, 1–16.

[CR15] O’Neill J (2016). Review on Antimicrobial Resistance: tackling a crisis for the health and wealth of nations. 2014.

[CR16] Oppong R, Jit M, Smith RD, Butler CC, Melbye H, Mölstad S, Coast J (2013). Cost-effectiveness of point-of-care C-reactive protein testing to inform antibiotic prescribing decisions. Br J Gen Pract.

[CR17] Wagner AK, Soumerai SB, Zhang F, Ross-Degnan D (2002). Segmented regression analysis of interrupted time series studies in medication use research. Journal of Clinical Pharmacy and Therapeutics.

[CR18] World Health Organization. (1996). *Guidelines for ATC classification and DDD assignment*.

[CR19] World Health Organization. (2015). *Global action plan on antimicrobial resistance*. 10.7196/samj.9644 26242647

